# Changes in state-level definitions of fetal death and the reporting of fetal death and periviable live births in the United States, 1996–2021: A panel study

**DOI:** 10.1016/j.annepidem.2026.110056

**Published:** 2026-02-17

**Authors:** Brenda Bustos, Allison Stolte, Alison Gemmill, Joan A. Casey, Dana Sarnak, Ralph Catalano, Tim A. Bruckner

**Affiliations:** a Department of Health, Society, and Behavior, Joe C. Wen School of Population and Public Health, University of California, Irvine, CA, USA; b Department of Sociology, University of Virgina, Charlottesville, VA, USA; c Department of Epidemiology, Fielding School of Public Health, University of California, Los Angeles, CA, USA; d Department of Environmental and Occupational Health Sciences, University of Washington, Seattle, WA, USA; e Department of Epidemiology, University of Washington, Seattle, WA, USA; f Department of Gynecology and Obstetrics, Johns Hopkins School of Medicine, Baltimore, MD, USA; g School of Public Health, University of California, Berkeley, Berkeley, CA, USA; h Center for Population, Inequality, and Policy, University of California, Irvine, CA, USA

**Keywords:** Fetal death definition, Fetal death reporting, Periviable births, Neonatal death

## Abstract

**Background:**

The World Health Organization’s 2030 Sustainable Development Goals include reducing the risk of fetal death. Even in high-income countries such as the United States (US), fetal deaths remain under-counted, with reporting showing variable quality across place and time. In the US, a uniform national definition of fetal death does not exist. Scant work characterizes whether, and to what extent, definitional changes in fetal death in US states over time affect fetal death reporting as well as counting of live births among similarly very frail (i.e., periviable [born <26 weeks]) infants. We aimed to (i.) identify state-level changes in fetal death reporting guidelines or definitions for all 50 US states from 1995 to 2020 and (ii.) examine whether counts of fetal deaths, periviable births, and neonatal deaths among periviable births shifted in the years following such changes.

**Methods:**

We retrieved data for all 50 US states from 1996 to 2021 for this descriptive analysis (n = 642,551 fetal deaths, n = 420,000 periviable births, n = 195,663 neonatal deaths among periviable births). We reviewed the fetal death user guides for state changes in reporting guidelines and conducted an internet search to find other state changes in the definition of fetal death. Next, we modeled fixed-effects linear regressions to examine associations between changes in fetal death reporting guidelines and our three outcomes.

**Results:**

Over the test period, 12 states changed their definition of fetal death. Regression results show increases in the counts of fetal deaths, periviable births, and neonatal deaths among periviable births following changes in reporting guidelines. These increases followed any change in reporting guidelines—whether perceived as a more inclusive or more restrictive change. Results hold across a range of alternative specifications.

**Conclusions:**

Our findings cohere with the notion that any state-level change in fetal death definitions corresponds with broader efforts to improve data collection and reporting protocols among not only fetal deaths but also periviable births. The fact that we observe such associations should encourage strategies to control for such “data breaks” for scientists and officials concerned with fetal death and/or periviable birth.

## Background

Reducing the risk of fetal death remains a key priority for numerous international organizations [1-3], such as the United Nations and World Health Organization, given its substantial toll on maternal and perinatal health. Many of the fetal deaths thought to be preventable worldwide occur in low- and middle-income countries [4]. However, even among high-income countries such as the United States (US), fetal death rates, particularly among some subgroups, remain high. For instance, despite recent decreases in the fetal mortality rate in the US, non-Hispanic (NH) Black mothers exhibit a two-fold greater likelihood of fetal mortality than do NH white mothers [5].

A recent Stillbirth Working Group, convened by the Eunice Kennedy Shriver National Institute of Child Health and Human Development (NICHD) Council, noted challenges with the consistency and quality of fetal death data collection across US states and counties [6]. The many data collection barriers highlighted by the Working Group indicate that fetal death counts in US vital statistics remain underreported. Reasons for this underreporting include the labor-intensive nature of completing the fetal death certificate form, lack of training and/or resources in some hospitals for filling out the form, and lack of state personnel and oversight in ensuring data collection on fetal deaths which lack a formal autopsy. These circumstances call into question the ability to compare fetal death rates across place and time and/or to derive accurate estimates of fetal death for subgroups (e.g., by race/ethnicity).

The accurate measurement of fetal death remains further complicated in the US because reporting guidelines vary across states. The 1992 Model Vital Statistics Law, produced by the U.S. National Center for Health Statistics, recommends reporting of fetal deaths with a birthweight of 350 g or greater or gestational age of 20 weeks or greater if birthweight is missing [7]. The US vital statistics 1997 revision in state definition reporting identifies a fetal death as “death prior to the complete expulsion or extraction from its mother of a product of human conception, irrespective of the duration of pregnancy and which is not an induced termination of pregnancy” [8]. The implementation of this definition, however, varies across states, with differences in gestational age and birthweight restrictions [7-9].

Variation in fetal death reporting may also affect measurement of live birth outcomes at the cusp of viability. Periviable births, born live at less than 26 complete (i.e., up to 25 6/7) weeks of gestation, account for 38 % of all infant deaths in the US [10]. Hospitals may erroneously classify some early neonatal deaths among this group as fetal deaths [11], potentially leading to mismeasurement of fetal and early neonatal death rates. On the other hand, prior work finds that states with birthweight-only guidelines under-report fetal deaths at lower gestational ages and over-report neonatal deaths at older gestations [9]. Based on this work, the extent to which reporting guidelines influence the counts of infant mortality, especially among births with limited viability [12], warrants further investigation.

We contribute to this small but important literature [13-15] by focusing on *within-state* changes over time in fetal death definitions and reporting guidelines. Specifically, (i.) we characterize, over a 25 + year period, the state-years in which these guidelines changed and (ii.) investigate whether formal changes in a state’s reporting guidelines affect counts of fetal deaths and periviable live births as well as the counts of neonatal death among periviable live births. The rationale for our descriptive analysis is two-fold. First, such changes may lead to either more inclusive or restrictive case definitions. Second, definitional changes may coincide with more attention (as well as resources) from state and hospital authorities to the fetal death undercounting problem than if the definitional change had not occurred.

## Methods

### Variables and Data.

Whereas the international community typically refers to pregnancy losses in the 2nd or 3rd trimester as stillbirths [16, 17], the US datasets use the term fetal deaths to refer to pregnancy loss at any time during pregnancy [18]. For this reason, we use the term fetal death when describing the data and analyses. We compiled changes in state-level fetal death definitions from two sources: the National Center for Health Statistics (NCHS) fetal death user guides and an internet search of documents from each of the state legislatures [19-27]. The NCHS-provided US fetal death user guides provide a table documenting the gestational age and/or birthweight criteria for reporting a fetal death for each state, since states determine reporting guidelines to be followed by clinicians and hospital staff within their jurisdiction. NCHS provides these guides for each year in our study period, allowing us to identify state- and year-specific reporting guidelines between 1995 and 2020. We reviewed the annual 1995–2020 fetal death user guides—each of which includes a table summarizing state-specific fetal death reporting requirements—noting changes from year to year in reporting guidelines. Specifically, we compared a guide’s table of reporting requirements in time t+1 to the table included in the prior years’ guide (time t) to identify changes in each state’s requirements for all study years (i.e., t = 1995 to t = 2020).

We then conducted an internet search for bills or legislation defining a change in the definition of fetal death for all 50 states. We utilized the Google search engine to search “[state name] fetal death definition change”. We only included changes in which the legislation identified the year of the change given the year was necessary to examine the change in counts. We identified definition or reporting changes in 12 states through these approaches. We were not able to find legislation information suggesting changes for all states. We therefore deemed the changes within the fetal death user guides to produce more reliable information on changes (vs. bills or legislation) given its availability for every state and year in our study period and conduct sensitivity analyses on only this subset of changes.

For the outcomes, we use restricted linked birth/infant death cohort and fetal death data from the 1996–2021 period, obtained from the NCHS, Division of Vital Statistics. The linked birth/infant death cohort files and fetal death files include data on gestational age, state, and year of birth/fetal death. The UC Irvine Committee for the Protection of Human Subjects approved this study (protocol # 20195444).

We include data for all 50 states. For the fetal death data, we restrict to fetal deaths occurring at or beyond 20 weeks of gestation. We do so given that (i.) only five states consistently report fetal death data prior to 20 weeks, and (ii.) much of the literature [16,28,29] focusing on causes and prevention of fetal death relate to events after 19 weeks, which typically involve a clinical visit [30]. Our fetal death outcome aggregates counts of fetal deaths by state and year (1996–2021). When examining periviable births, we restrict to live births occurring between 20 and < 26 (i.e., up to 25 6/7 weeks) completed weeks of gestation [31]. We aggregate the counts of periviable births and separately neonatal deaths (occurring 0–27 days after live birth) among periviable births by state and year. Additionally, as a robustness check, we aggregate counts of all births to calculate (a) total live births plus fetal deaths, (b) total live births only, and (c) total periviable births to calculate rates of our main outcomes. The 1996–2021 time span represents the longest range of data, with consistent classification of covariates, available to us at the time of our tests.

We examine state’s fetal death definitions over the study period (1996–2021), compared to their definitions in the year prior. We categorized definition changes as more restrictive/likely to decrease fetal death reporting (−1), more inclusive/likely to increase fetal death reporting (1), or neutral/unlikely to increase or decrease fetal death reporting/no change (0). States with a definition change in language only, coded as a 1, indicate that different clinical manifestations than the previous definition were included in the definition and that there were no changes in the gestational age and/or birthweight cutpoints in the fetal death user guide. We coded the one-word change in the definitions for Indiana and Illinois as 0 given the lack of description of clinical manifestations that could be compared to the previous definition. We also assigned neutral/no change (0) to all state-years between 1996 (the first year we assess “change”) and the year prior to a state’s first definition change (or to all years, if a state never implemented a change). When a state updated a definition, we assigned a change measure for that year and all following years depending on the nature of the change. We then lagged the change data in our analysis to consider the change in outcomes in the following t + n years’ reporting. For instance, guidelines moving the minimum birthweight of a record from 500 g to 350 g would constitute a more inclusive fetal death reporting guideline, and we would assign a change value of 1 for the year of the change and all future years. By contrast, limiting records to birthweights greater than 350 g or beyond 20 weeks of gestation (vs. guidelines with no weight or week restriction) would, in our view, constitute more restrictive guidelines. Neutral guidelines relate to changes only in terminology, such as “mother” to “uterus”.

### Statistical Analyses.

Our three outcomes are: (i.) fetal death counts; (ii.) periviable live birth counts; and (iii.) counts of neonatal deaths (i.e., death of a live birth before 28 days) among periviable live births. As a robustness check, we also examined (i.) the rate of fetal deaths per 1000 total live births and fetal deaths; (ii.) the rate of periviable live births per 1000 live births; and (iii.) the rate of neonatal death among periviable births per 100 periviable births.

We reason that a state’s change in fetal death reporting guidelines in a particular year t could affect fetal death counts in state-years t+n, such that n ranges from 1 to the number of years until either the end of the study period (2021) or the next definition change. In this step function approach, the definitional change renders as exposed all years including and following the year of the definitional change.

We utilize linear regression with a 1-year lagged state and year fixed effects to control for both state-level differences in reporting and for any broader US trends over time in reporting that are shared across all states. This strategy allows for assessment of global trends in the outcome (i.e. fetal death counts, neonatal death counts, neonatal deaths among periviable births) while allowing for within-state change to vary and contribute to the estimate for global trends. This fixed effect strategy is increasingly used in epidemiology [32], given the noted inferential limitations of cross-sectional work which compares health outcomes across place (i.e., state), but cannot adequately control for the fact that states are non-exchangeable [33].

Given efforts to improve viability of infants at earlier gestational ages [34-37], changes in fetal death reporting guidelines may contribute to either increases or decreases in reporting of outcomes beyond fetal deaths but at the cusp of viability. Whereas we have no strong prior about whether, and how, changes in fetal death reporting guidelines could affect the count of periviable live births as well as neonatal deaths among periviables, we similarly assumed (as with the fetal death counts) that reporting guideline changes in year *t* could precede changes in these counts in years t+1…n as well. We therefore examine associations of fetal death reporting changes with the count of periviable births and with the risk of neonatal death among periviable births. As with the test of fetal death counts, we conduct these analyses within a state and year fixed-effects regression framework in which the unit of analysis is the state-year.

Changes in fetal death reporting definitions—whether or not they are believed to expand the scope of cases considered to fall under that definition—may on their own reflect elevated awareness (or resources) by state or hospital authorities to vital statistics reporting practices. Given this possibility, we also examined whether *any* definitional change (either more inclusive or more restrictive) in year t is associated with that state’s increase in outcome counts in year t and all following years. Additionally, as a falsification test, we examine whether fetal death reporting definition changes are associated with counts of periviable births that do not end in infant mortality. We do so given that changes in how a fetal death is reported should not influence the count of periviable births that survive and thrive. All analyses were conducted using STATA V17.0.

## Results

Between 1995 and 2020, 12 states changed fetal death reporting definitions. Table 1 lists the year of the legislative change for these 12 states and the states’ original and revised reporting guidelines for fetal death. We note 14 changes across 12 states—Arkansas and New Mexico experienced two changes in reporting during our study period (both no change to more restrictive and then more restrictive to more inclusive). Reporting guidelines include changes in gestational age range, birthweight cutpoints, or clinical symptoms for which fetal death may be indicated. Illinois and Indiana exhibit neutral changes that relate to terminology only. We categorized eight changes as more inclusive and potentially leading to greater counts of fetal deaths in the subsequent year. We also categorized four changes as more restrictive and potentially leading to fewer counts of fetal deaths in the subsequent year.

**Fig. 1.**
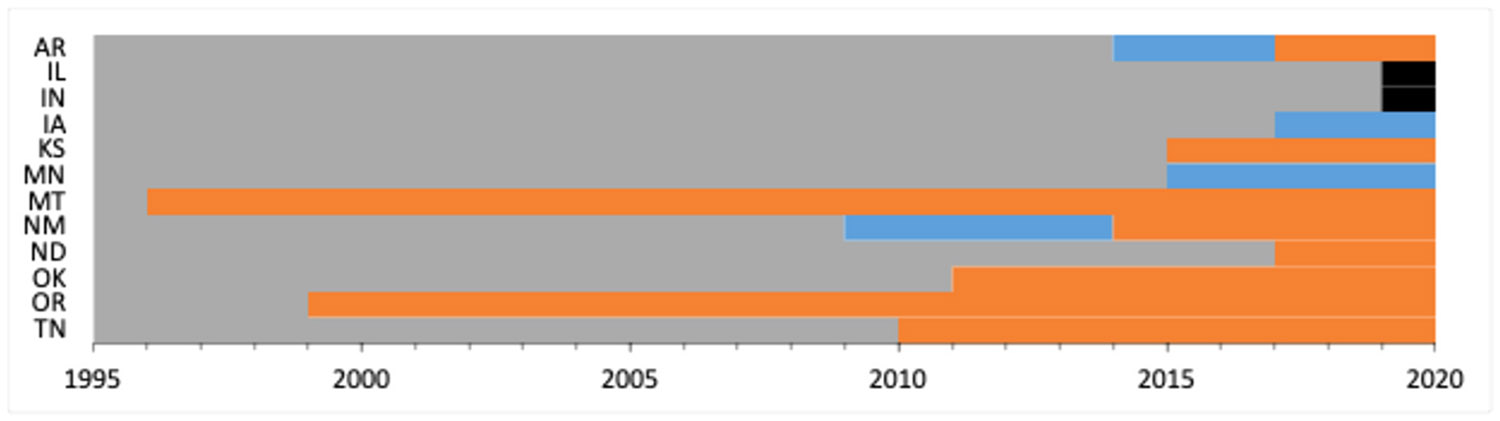
States with fetal death reporting guideline or fetal death definition changes between 1995 and 2020. Light blue denotes states with more restrictive changes (−1). Orange denotes states with more inclusive changes (+1). Black denotes states with neutral changes (0). States with more than one color indicate more than one change occurred in the study period.

Fig. 1 plots the timing of the fetal death reporting definition change for the 14 states that showed a change. All but 3 of the 12 states (Montana, New Mexico, and Oregon) exhibit at least one definition change after 2010.

Table 2 provides summary statistics of the three outcome variables for all states over the test period. States with changes include states with both high counts of births (e.g., Illinois) and low counts of births (e.g., Montana). Supplemental Figure 1 shows state-specific plots over time of the outcomes studied, as well as color-coded indications of state-years experiencing a change in reporting definitions (note variation in y-axes across states). Tennessee, for instance, appears to show a large increase in fetal death counts following its inclusive reporting change.

Table 3 shows our main results from the fixed effects regression approach (n = 1300 state-years for fetal deaths, periviable births, and neonatal deaths among periviable births). We find a positive association between more inclusive definitional changes in year t and counts of fetal deaths (i.e., coef: 113.21 additional fetal deaths, standard deviation [SD] = 15.71, p < .01), periviable live births (i.e., coef: 29.19, SD = 8.92, p < 0.01), and neonatal deaths among periviable live births (i.e., coef: 24.46, SD = 5.44, p < 0.01) in years t+n in that state. In comparison, we find only about 56 counts greater than expected (SD = 22.83, p < 0.10) fetal deaths for state years following more “restrictive” guidelines. Additionally, we find detectable increases in periviable birth counts and neonatal death counts among periviables in the state years following the implementation of more restrictive guidelines. The increases in periviable births and neonatal deaths are similar than those observed among state-years with more inclusive guidelines.

We then, as a robustness check, examined rates of fetal death and periviable birth outcomes rather than count outcomes. Inference for all three rate outcomes remains similar to the count outcomes reported in Table 3 (Supplemental Table 1) in that we observe statistically detectable increases in all three rates in years following states’ more inclusive definitional changes in fetal death. The magnitude of the rate increase appears largest for the rate of neonatal death among periviable births.

Given the possibility that any change to fetal death definitions may indicate a broader interest in revising data collection protocols or heightened state oversight of hospitals, we then considered *any definitional change* in fetal death at year t as the exposure of interest. Regression results again indicate statistically detectable increases in fetal deaths, periviable births, and neonatal deaths among periviable births in years t+n (all results are statistically detectable at p < .01 level [Table 4]), with effect sizes comparable to inclusive changes in definitions. Similarly, when examining rates rather than counts, effects sizes remain comparable to inclusive changes in definitions (Supplemental Table 2).

We performed three additional robustness checks to assess sensitivity of results to alternative specifications. First, given that Tennessee shows a disproportionately large increase in fetal death counts and has a large base count of fetal deaths, we assessed whether main regression results hold after excluding Tennessee from the analysis to ensure the state’s patterns did not drive our main results. The coefficient for more inclusive definitional changes in our main fetal death model becomes attenuated by ~33 % but remains statistically detectable; results for periviable births and neonatal deaths remain similar to the original test (right columns of Table 3 [and Supplemental Table 1]). Second, we recoded the reporting change exposure variable to include only changes identified within the reporting criteria for fetal deaths found in the NCHS user guides (N = 6). In other words, we did not consider fetal death definition changes identified from an internet search of state legislation. The changes from the state legislation relay changes in language in the fetal death definition rather than changes in reporting criteria. These additional analyses produced similar findings to the original tests, particularly for the more inclusive changes (Supplemental Tables 3-6). Lastly, as a falsification check we examined the counts of periviable births that do not end in death within 365 days of live birth as an outcome (Supplemental Table 7). We did so given that changes in what defines a fetal death should not influence the number of periviable live births that survive past age one. In other words, the births could never be misclassified as a fetal death that was or was not reported. As expected, we do not find any relation between definitional changes and the counts of periviable births that do not end in infant death.

## Discussion

Several federal and international workgroups note under-reporting of fetal death counts in both low- and high-income countries [4,13,15]. This under-reporting, as well as inconsistent reporting protocols across place and time, make it challenging to conduct basic epidemiological studies on the causes of fetal death. We set out to determine the relation, if any, between state-level changes in fetal death reporting guidelines in the US and subsequent counts of fetal deaths, periviable live births, and neonatal deaths among periviable live births. Using vital statistics data from all 50 states from 1996 to 2021, we find detectable increases in counts following state-level changes in the reporting guideline for fetal deaths. Interestingly, the count of periviable births as well as neonatal deaths among periviable births also increases in years after definitional changes in fetal deaths. Our findings suggest that changes in fetal death reporting guidelines may reflect broader state-level efforts that improve data collection protocols of vital perinatal events. Importantly, they also suggest that studies should account for definitional changes over time when examining fetal death trends, as well as differences in definitions when comparing fetal death rates across different reporting entities.

Strengths of our study include utilizing state and year fixed effects to control for unobserved factors that may confound the relation between changing fetal death definitions and our outcomes of interest [38]. This strategy accounts for unique characteristics of a state that may influence reporting, such as political-legal environment or level of resources allocated to data collection that vary substantially across state contexts. In addition, results cannot arise due to broader US-wide trends in fetal death reporting because we controlled for time-trends in all analyses. Lastly, the robustness and falsification checks we performed enhance the likelihood that results do not arise due to chance.

Our study is not without limitations. The US fetal death user guides do not provide the written individual state definitions. Rather, the guides list a summary table of the reporting guidelines for each state and note any year-to-year changes. Additionally, we cannot directly determine whether the practices of clinicians or hospitals change after a fetal death definition change [11,39]. Third, by utilizing states as our geographic region, we cannot determine whether the practice of reporting of fetal deaths varies within the state or by clinical or demographic subgroup. We therefore encourage state-specific examinations that assess the relation between reporting guidelines and birth outcomes. Such work could especially benefit from careful scrutiny of states with rather large and abrupt changes in fetal death counts, as indicated by state-specific plots in Supplemental Figure 1 (e.g., Kansas and Tennessee).

Although we coded certain definitional changes as “more restrictive,” we observed only positive associations between these state-year changes and subsequent fetal death counts. These findings, however, did not extend to models that excluded legislative definitional changes. We suspect that these results may arise due to any definitional change, and especially any legislatively dictated change, reflecting broader attention by state authorities to the undercounting challenge of vital perinatal events, especially given that we also observed increased counts of periviable births and neonatal deaths. We note, however, that evidence in this area remains sparse; for this reason, we acknowledge that our interpretation remains only informed speculation at this point. In addition, given the time frame studied, our work cannot inform the important questions about whether the post-*Dobbs* abortion policy landscape, which involve the Supreme Court’s decision to remove the federal constitutional right to abortion in June 2022 [40], could affect counts of fetal death, periviable birth, and neonatal deaths among periviable births. That stated, other work supports the notion that the “right-to-life” debate leading up to the *Dobbs* decision has stimulated public interest in the measures we study [41-43].

Theories of organizational behavior [44] as well as policy feedback [45] provide a plausible framework for our findings. For instance, shifts in fetal death reporting guidelines could impose new normative and/or regulatory expectations on hospitals that compel workers to more thoroughly report vital events. Alternatively, policy feedback theory contends that the adoption of policies (in our case, of a new guideline for fetal death reporting) could coincide with more resources and incentives for hospitals to more accurately collect vital event data [46]. Any of these pathways could plausibly affect not only fetal death counts but also recording of neonatal deaths among periviable births, who are increasingly saved at earlier gestational ages due to advances in medical technology [47]. Whereas data limitations precluded a more careful examination of the extent to which these theories apply to the vital records landscape, we encourage such additional research regarding potential mechanisms for our observed findings.

## Conclusion

Our investigation supports and extends previous work suggesting potential under- or over-reporting of counts of neonatal death [9]. In addition, our work aligns with recent findings in Quebec in which recorded stillbirth rates rose following a more inclusive minimum gestational age definition of fetal death [13]. At a minimum, results showing increases in counts of fetal deaths, periviable births, and neonatal death among periviable births following a change in fetal death definition or reporting guideline should compel researchers using US vital statistics data to control for administrative “breaks” in reporting when attempting to identify causal antecedents of fetal death. We encourage a collaborative effort between policy and practice to elucidate the contexts surrounding such “breaks” and pro-actively track future changes over time. In addition, future work should examine whether, and how, reporting guidelines affect the observed incidence of live birth outcomes. Such information could elucidate whether definitional changes in reporting bias our understanding of infant health disparities.

## Supplementary Material

1

Supplementary data associated with this article can be found in the online version at doi:10.1016/j.annepidem.2026.110056.

## Figures and Tables

**Table 1:** States with any change in fetal deaths reporting guidelines or definitions. Changes determined between 1995 and 2020^†^.

	Initial reporting guideline	Reporting or definition change	Direction of expected change related to fetal death reporting
**Arkansas**	All periods of gestational age	2014: 350 g or 20 weeks if weight unknown2017: 12 weeks	−1*,1*
**Illinois**	Gestational age of at least 20 weeks	2019: changed from 'its mother' to 'a uterus'	0
**Indiana**	Gestational age of at least 20 weeks	2019: changed from 'complete birth' to 'live birth'	0
**Iowa**	Gestational age of at least 20 weeks	2017: added 'heartbeats shall be distinguished from transient cardiac contractions, and respirations shall be distinguished from fleeting respiratory efforts or gasp'	−1
**Kansas**	Birthweight of at least 350 g	2015: 20 weeks	1*
**Minnesota**	Gestational age of at least 20 weeks	2015: added 'heartbeats shall be distinguished from transient cardiac contractions, and respirations shall be distinguished from fleeting respiratory efforts or gasp'	−1
**Montana**	Gestational age of at least 20 weeks or birthweight of at least 500 g	1996: 350 g or 20 weeks if weight unknown	1*
**New Mexico**	Birthweight of at least 500 g	2009: added 'results in other than a live birth and that is not an induced abortion' 2014: 20 weeks or 350 g	−1, 1*
**North Dakota**	Gestational age of at least 20 weeks	2017: removed irrespective of duration of pregnancy	1
**Oklahoma**	Gestational age of at least 20 weeks	2011: 12 weeks	1*
**Oregon**	Gestational age of at least 20 weeks	1999: added irrespective of duration and death indications	1
**Tennessee**	Birthweight of at least 500 g	2010: 20 weeks or 350 g	1*

* Indicates changes within the fetal death user guides.

† Changes are 1 = more inclusive/predicted increase, −1 = more restrictive/predicted decrease, and 0 =neutral or no change.

**Table 2:** Annual mean and range of fetal deaths, periviable births, and neonatal deaths among periviable births between 1996 and 2021.

State	Fetal deaths		Periviable births		Neonatal deaths among periviable births	
	Mean(Std Dev)	Range	Mean(Std Dev)	Range	Mean(Std Dev)	Range
Alabama	533 (31)	486–584	365 (40)	272–421	152 (36)	83–223
Alaska	50 (10)	36–72	29 (7)	19–47	10 (4)	3–22
Arkansas*	262 (29)	207–312	155 (14)	123–181	67 (10)	54–89
Arizona	512 (51)	422–611	290 (40)	236–376	144 (27)	91–190
California	2747 (338)	2190–3364	1496 (148)	1179–1820	707 (85)	527–856
Colorado	375 (23)	329–432	213 (28)	165–259	122 (23)	78–165
Connecticut	217 (44)	139–299	170 (33)	118–229	90 (26)	47–144
Delaware	62 (9)	43–82	65 (11)	48–86	35 (11)	14–56
Florida	1585 (78)	1461–1817	1014 (87)	835–1193	469 (56)	362–585
Georgia	1132 (83)	985–1285	684 (70)	572–837	315 (60)	198–395
Hawaii	115 (13)	92–138	69 (12)	47–86	36 (8)	20–52
Iowa*	196 (24)	144–240	116 (11)	96–135	49 (14)	25–79
Idaho	112 (8)	97–130	51 (11)	29–76	30 (8)	14–46
Illinois*	1026 (166)	732–1307	754 (136)	422–930	376 (87)	199–518
Indiana*	516 (50)	424–612	337 (34)	255–413	171 (27)	115–219
Kansas*	192 (23)	143–267	139 (16)	105–176	70 (11)	47–93
Kentucky	343 (23)	293–383	203 (16)	166–228	75 (11)	57–102
Louisiana	393 (83)	270–532	405 (48)	305–487	146 (34)	91–208
Massachusetts	373 (55)	285–457	256 (47)	154–321	130 (33)	60–175
Maryland	542 (66)	426–696	369 (37)	278–423	184 (30)	126–232
Maine	64 (10)	41–85	42 (7)	32–57	25 (6)	17–42
Michigan	674 (79)	540–821	570 (79)	407–657	282 (62)	185–373
Minnesota*	365 (20)	332–400	220 (22)	182–270	97 (14)	73–132
Missouri	456 (32)	396–512	345 (37)	264–413	164 (32)	106–218
Mississippi	405 (40)	346–501	230 (36)	175–301	102 (20)	66–135
Montana*	52 (9)	37–79	31 (7)	20–50	14 (4)	8–22
North Carolina	828 (65)	659–937	655 (52)	550–751	320 (46)	222–388
North Dakota*	62 (11)	44–84	36 (7)	21–49	16 (3)	11–22
Nebraska	146 (19)	100–176	85 (12)	65–106	45 (10)	31–72
New Hampshire	62 (7)	48–75	31 (9)	18–54	17 (5)	8–28
New Jersey	703 (44)	590–778	441 (81)	277–588	196 (53)	96–287
New Mexico	84 (15)	58–122	74 (13)	50–98	35 (7)	21–47
Nevada	230 (36)	160–282	115 (27)	60–161	50 (14)	21–86
New York	1977(402)	1287–2617	969 (158)	687–1246	449 (110)	254–686
Ohio	927 (83)	761–1150	670 (63)	503–807	333 (45)	213–397
Oklahoma*	288 (27)	239–337	192 (29)	137–245	86 (17)	54–130
Oregon*	228 (24)	183–271	116 (16)	78–139	70 (13)	43–94
Pennsylvania	986 (147)	670–1265	634 (68)	485–758	330 (51)	216–409
Rhode Island	69 (10)	52–89	63 (14)	39–98	33 (9)	15–50
South Carolina	459 (78)	297–573	290 (35)	225–369	138 (31)	88–191
South Dakota*	61 (20)	34–98	40 (8)	25–57	19 (6)	9–31
Tennessee*	497 (101)	356–674	413 (37)	347–482	190 (38)	113–250
Texas	1953 (203)	1500–2247	1523 (174)	1120–1758	577 (67)	433–670
Utah	272 (33)	214–327	142 (16)	108–175	75 (11)	51–94
Virginia	652 (99)	468–812	453 (40)	388–545	211 (35)	148–274
Vermont	27 (5)	16–38	16 (5)	8–27	10 (4)	3–18
Washington	484 (42)	407–588	218 (27)	165–264	106 (13)	85–131
Wisconsin	357 (32)	300–412	239 (27)	177–285	127 (16)	89–161
West Virginia	122 (25)	76–169	83 (10)	65–101	40 (8)	25–61
Wyoming	32 (6)	22–47	9 (3)	4–17	6 (2)	4–9

* Denotes states with a definition change between 1995 and 2020.

**Table 3:** State and year fixed effects linear regression models predicting counts of perinatal outcomes following inclusive or restrictive reporting changes*.

	Model 1 (N = 1300)	Model 2^†^ (N = 1274)
	Coef (Std Error)	Coef (Std Error)
**Fetal Deaths**		
**More Inclusive vs. No Change**	113.21 (15.71)***	76.18 (17.34)***
More Restrictive vs. No Change	56.43 (22.83)*	51.91 (22.83)**
**Periviable Births**		
More Inclusive vs. No Change	29.19 (8.92)***	37.03 (9.91)***
More Restrictive vs. No Change	34.95 (12.97)**	36.09 (13.04)***
**Neonatal Deaths among Periviables**		
More Inclusive vs. No Change	24.46 (5.44)***	36.68 (5.99)***
More Restrictive vs. No Change	25.59 (7.90)***	27.19 (7.88)***

p < 0.10*; p<0.05**; p<0.01***

* Models examine the relation between any reporting changes with more inclusive/predicted increases (=1), more restrictive/predicted decreases (= −1), no change/neutral changes (=0) and counts of outcomes in the subsequent years. Reporting changes measured between 1995 and 2020. Counts of each outcome measured between 1996 and 2021. Model 1 includes all 50 states. Model 2 excludes Tennessee

† We exclude Tennessee in model 2 given a large increase in fetal deaths after a reporting change to assess whether findings remain robust after removing Tennessee.

**Table 4:** State and year fixed effects linear regression models predicting counts of perinatal outcomes following any changes*.

	Model 1 (N = 1300)	Model 2^†^ (N = 1274)
	Coef (Std Error)	Coef (Std Error)
**Fetal Deaths**		
Any Change vs. No Change	96.41 (13.73)***	67.80 (14.69)***
**Periviable Births**		
Any Change vs. No Change	30.90 (7.78)***	36.71 (8.39)***
**Neonatal Deaths among Periviables**		
Any Change vs. No Change	24.80 (4.74)***	33.40 (5.07)***

p < 0.10*; p<0.05**; p<0.01***

* Models examine the relation between *any reporting change* and *counts* of three different outcomes in the subsequent years following the change. Reporting changes measured between 1995 and 2020. Counts of each outcome measured between 1996 and 2021. Model 2 excludes Tennessee.

† We exclude Tennessee in model 2 given a large increase in fetal deaths after a reporting change to assess whether findings remain robust after removing Tennessee.

## Data Availability

The data that support the findings of this study are available from NCHS Vital Statistics Division but restrictions apply to the availability of these data.
