# Supplementary material for: Changes in state-level definitions of fetal death and the reporting of fetal death and periviable live births in the United States, 1996–2021: A panel study
Source: Ann Epidemiol. Author manuscript; Available in PMC 2026 Jun 7. (PMC13242943; doi:10.1016/j.annepidem.2026.110056)
Supplement: 1 [file NIHMS2178252-supplement-1.pdf]

SUPPLEMENTAL MATERIAL

Supplemental Table 1. Rates of outcomes. State and year fixed effects linear regression models[1,2].

	Model 1 (N=1,300) Coef (Std Error)	Model 2 (N=1,274) Coef (Std Error)
**Fetal Deaths per 1,000 Total Births**		
More Inclusive vs. No Change	0.86 (0.12)***	0.46 (0.14)***
More Restrictive vs. No Change	0.06 (0.18)	0.01 (0.18)
**Periviable Births per 1,000 Live Births**		
More Inclusive vs. No Change	0.17 (0.08)**	0.27 (0.09)***
More Restrictive vs. No Change	0.35 (0.11)***	0.37 (0.11)***
**Neonatal Deaths among Periviables per 100 Periviable Births**		
More Inclusive vs. No Change	3.22 (1.20)***	5.26 (1.33)***
More Restrictive vs. No Change	1.16 (1.75)	1.43 (1.76)

p<0.10*, <0.05**, <0.01***

[1] Models examine the relation between any reporting changes with more inclusive/predicted increases (=1), more restrictive/predicted decreases (= -1), no change/neutral changes (=0) and rates of outcomes in the subsequent years. Reporting changes measured between 1995 to 2020. Rates of each outcome measured between 1996 to 2021. Model 1 includes all 50 states. Model 2 excludes Tennessee.

[2]We exclude Tennessee in model 2 given a large increase in fetal deaths after a reporting change to assess whether findings remain robust after removing Tennessee.

Supplemental Table 2. Rates of outcomes. State and year fixed effects linear regression models[1,2].

	Model 1 (N=1,300) Coef (Std Error)	Model 2 (N=1,274) Coef (Std Error)
**Fetal Deaths per 1,000 Total Births**		
Any Change vs. No Change	0.62 (0.11)***	0.31 (0.12)***
**Periviable Births per 1,000 Live Births**		
Any Change vs. No Change	0.22 (0.07)***	0.30 (0.73)***
**Neonatal Deaths among Periviables per 100 Periviable Births**		
Any Change vs. No Change	2.61 (1.05)**	3.94 (1.13)***

p<0.10*, <0.05**, <0.01***

[1]Models examine the relation between *any reporting change* and *rates* of three different outcomes in the subsequent years following the change. Reporting changes measured between 1995 to 2020. Rates of each outcome measured between 1996 to 2021. Model 2 excludes Tennessee.

[2]We exclude Tennessee in model 2 to assess whether the large increase in fetal deaths after a reporting change in Tennessee drive the overall results.

Supplemental Table 3. State and year fixed effects linear regression examining changes(-1 or 1) in fetal death user guide only[1,2].

	**Coef. (Std Error) N=1,300**
**Fetal Deaths**	
More Inclusive vs. No Change	119.54 (17.40)***
More Restrictive vs. No Change	15.72 (53.44)
**Periviable Births**	
More Inclusive vs. No Change	25.59 (9.89)**
More Restrictive vs. No Change	19.25 (30.37)
**Neonatal Deaths among Periviables**	
More Inclusive vs. No Change	19.45 (6.05)***
More Restrictive vs. No Change	13.46 (18.57)

p<0.10*, <0.05**, <0.01***

[1] Models examine the relation between *step* reporting changes and *counts* of outcomes in the subsequent year among all births accounting for predicted increases =1 and decreases =-1 following the change. Accounting for differences in reporting guideline changes noted in the fetal death user guides only.

[2]No changes in the fetal death definition from legislation included. Excludes the following changes: Iowa 2017, Minnesota 2015, New Mexico 2009, North Dakota 2017, and Oregon 1999.

Supplemental Table 4. State and year fixed effects linear regression examining changes(-1 or 1) in fetal death user guides only[1,2].

	Coef. (Std Error) N=1,300
**Fetal Deaths per 1,000 Total Births**	
More Inclusive vs. No Change	0.99 (0.14)***
More Restrictive vs. No Change	-0.76 (0.42)*
**Periviable Births per 1,000 Live Births**	
More Inclusive vs. No Change	0.17 (0.09)*
More Restrictive vs. No Change	0.11 (0.26)
**Neonatal Deaths among Periviables per 100 Periviable Births**	
More Inclusive vs. No Change	2.52 (1.33)*
More Restrictive vs. No Change	-0.70 (4.09)

p<0.10*, <0.05**, <0.01***

[1] Models examine the relation between *step* reporting changes and *rates* of outcomes in the subsequent year among all births accounting for predicted increases =1 and decreases =-1 following the change. Accounting for differences in reporting guideline changes noted in the fetal death user guides only.

[2]No changes in the fetal death definition from legislation included. Excludes the following changes: Iowa 2017, Minnesota 2015, New Mexico 2009, North Dakota 2017, and Oregon 1999.

Supplemental Table 5. State and year fixed effects linear regression examining changes(=1) in fetal death user guides only[1,2].

	Coef. (Std Error) N=1,300
**Fetal Deaths**	
Any Change vs. No Change	111.52 (16.89)***
**Periviable Births**	
Any Change vs. No Change	25.10 (9.59)***
**Neonatal Deaths among Periviables**	
Any Change vs. No Change	18.98 (5.86)***

p<0.10*, <0.05**, <0.01***

[1] Models examine the relation between *step* reporting changes and *counts* of outcomes in the subsequent year among all births accounting for predicted increases =1 and decreases =-1 following the change. Accounting for differences in reporting guideline changes noted in the fetal death user guides only.

[2]No changes in the fetal death definition from legislation included. Excludes the following changes: Iowa 2017, Minnesota 2015, New Mexico 2009, North Dakota 2017, and Oregon 1999.

Supplemental Table 6. State and year fixed effects linear regression examining changes(=1) in fetal death user guides only[1,2].

	Coef. (Std Error) N=1,300
**Fetal Deaths per 1,000 Total Births**	
Any Change vs. No Change	0.85 (0.13)***
**Periviable Births per 1,000 Live Births**	
Any Change vs. No Change	0.17 (0.08)**
**Neonatal Deaths among Periviables per 100 Periviable Births**	
Any Change vs. No Change	2.27 (1.29)*

p<0.10*, <0.05**, <0.01***

[1] Models examine the relation between *step* reporting changes and *rates* of outcomes in the subsequent year among all births accounting for predicted increases =1 and decreases =-1 following the change. Accounting for differences in reporting guideline changes noted in the fetal death user guides only.

[2]No changes in the fetal death definition from legislation included. Excludes the following changes: Iowa 2017, Minnesota 2015, New Mexico 2009, North Dakota 2017, and Oregon 1999.

Supplemental Table 7. State and year fixed effect linear regression models examining counts of surviving periviable births[1].

	**Coef. (Std Error) N=1,300**
**Fetal deaths**	
More Inclusive vs. No Change	2.04 (5.11)
More Inclusive vs. No Change	5.70 (7.42)
Any Change vs. No Change	3.12 (4.46)

p<0.10*, <0.05**, <0.01***

[1] Falsification test to examine the relation between ***step*** reporting changes and the counts of periviable live births that do not end in infant death in the subsequent years following definitional changes.

Supplemental Figure 1. State-specific plots of counts of (A) fetal death, (B) periviable birth, and (C) neonatal death among periviable births.

A

B

C

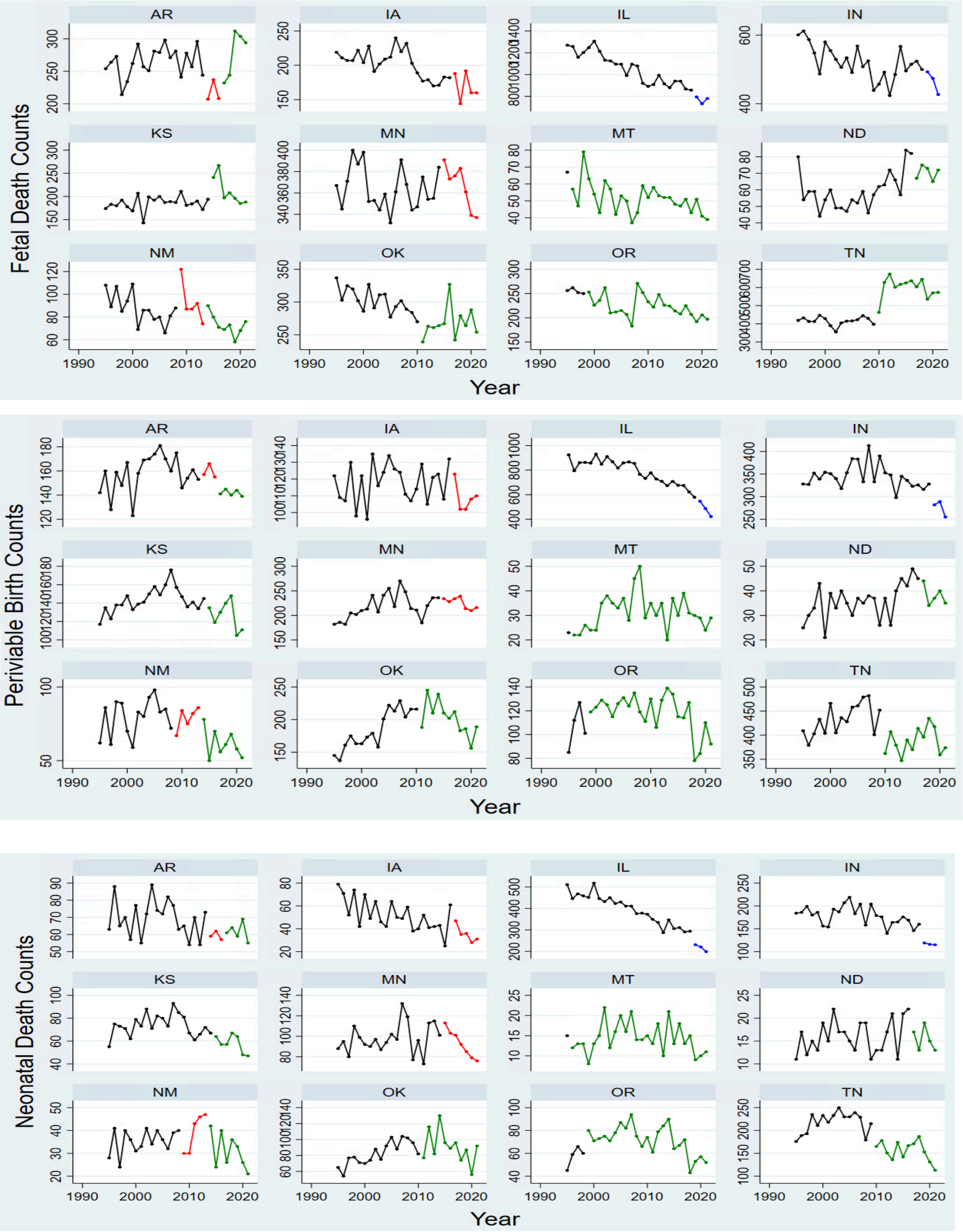

[1]Plots for 14 states exhibiting neutral (**blue**), inclusive (**green**), and restrictive (**red**) definition or reporting guideline changes between 1995 to 2021.
